# COVID-19-Associated Pulmonary Aspergillosis: A Single-Center Experience in Central Valley, California, January 2020–March 2021

**DOI:** 10.3390/jof7110948

**Published:** 2021-11-10

**Authors:** Geetha Sivasubramanian, Hebah Ghanem, Michele Maison-Fomotar, Ratnali Jain, Robert Libke

**Affiliations:** 1 Division of Infectious Diseases, Department of Internal Medicine, University of California, San Francisco, CA 93701, USA; hebah.ghanem@ucsf.edu (H.G.); micmam5@yahoo.com (M.M.-F.); robert.libke@ucsf.edu (R.L.); 2 UCSF Fresno Research Institute, University of California, San Francisco, CA 93701, USA; ratnali.jain@ucsf.edu

**Keywords:** Covid-19, invasive pulmonary aspergillosis, CAPA

## Abstract

Reports of coronavirus disease 2019 (COVID-19)-associated pulmonary aspergillosis (CAPA) have been widely published across the world since the onset of the pandemic with varying incidence rates. We retrospectively studied all patients with severe COVID-19 infection who were admitted to our tertiary care center′s intensive care units between January 2020 and March 2021, who also had respiratory cultures positive for *Aspergillus* species. Among a large cohort of 970 patients admitted to the ICU with severe COVID-19 infections during our study period, 48 patients had *Aspergillus* species growing in respiratory cultures. Based on the 2020 European Confederation of Medical Mycology and the International Society for Human and Animal Mycology (ECMM/ISHAM) consensus criteria, 2 patients in the study had proven CAPA, 9 had probable CAPA, and 37 had possible CAPA. The incidence of CAPA was 5%. The mean duration from a positive COVID-19 test to *Aspergillus* spp. being recovered from the respiratory cultures was 16 days, and more than half of the patients had preceding fever or worsening respiratory failure despite adequate support and management. Antifungals were given for treatment in 44% of the patients for a mean duration of 13 days. The overall mortality rate in our study population was extremely high with death occurring in 40/48 patients (83%).

## 1. Introduction

Invasive pulmonary aspergillosis (IPA) is a devastating infection associated with high morbidity and mortality. IPA was traditionally encountered in the immunocompromised host, typically patients with prolonged neutropenia, hematologic malignancy, allogeneic hematopoietic stem cell or solid organ transplantation, prolonged use of high dose corticosteroids and T-cell immunosuppressive agents [1]. In the past decade, evidence started to accumulate that IPA also occurred in patients admitted to the intensive care unit (ICU) who did not otherwise have the classic host factors [2]. A cohort of these ICU patients had preceding respiratory viral illnesses such as influenza [3,4]. IPA was already a challenging diagnosis in patients with traditional risk factors due to the difficulty of obtaining tissue samples to confirm diagnosis. IPA in post-viral illness and critically ill patients is even more difficult to diagnose due to the absence of characteristic clinical and imaging findings. Clinical algorithms have been designed specifically to define IPA in this context [5,6].

Since the onset of the severe acute respiratory syndrome coronavirus 2 (SARS-CoV-2)-associated pandemic, multiple reports of IPA have emerged in critically ill patients with severe pneumonia [7,8,9,10,11,12,13,14,15,16,17,18,19,20,21,22,23,24,25]. The incidence of COVID-19-associated pulmonary aspergillosis (CAPA) in these studies has been variable, ranging from 3 to 34% largely due to differences in diagnostic criteria as well as regional differences in obtaining culture specimens and bronchoscopies [14,26]. Expert consensus definition criteria for CAPA have been published [27]. We report our experience on CAPA among patients with severe COVID-19 infection in Fresno, Central California, from January 2020 through March 2021.

## 2. Materials and Methods

The study was conducted in a tertiary care center located in Fresno, Central California, United States of America with 685 beds and 55 intensive care unit beds. During the surge of cases, the number of ICU beds was expanded up to two or three times the normal capacity, averaging between 85 and 150 ICU beds.

We conducted a retrospective analysis of patients who were admitted to the intensive care unit with COVID-19 infection and had *Aspergillus* species growing from their respiratory cultures. All patients above the age of 18 who were admitted to the intensive care unit between January 2020 and March 2021, who had a confirmed SARS-Cov-2 PCR test and had positive *Aspergillus* cultures from subsequent respiratory tract specimens were included in the study. Data were collected from the patient charts including demographics, underlying medical conditions, clinical features, length of stay, mortality, radiological and laboratory findings as well as treatments. We also compared the total number of positive *Aspergillus* respiratory cultures in the intensive care unit patients between January 2020 and March 2021 with the previous two years of positive *Aspergillus* respiratory cultures in the intensive care unit patients. COVID-19-associated pulmonary aspergillosis (CAPA) was defined using the 2020 ECMM/ISHAM consensus criteria and categorized as proven, probable, and possible.

### 2.1. Laboratory Methods

Patients were identified as positive for COVID-19 by having a positive polymerase chain reaction (PCR) for SARS-CoV-2 from nasopharyngeal swab, endotracheal aspirate or bronchoalveolar lavage specimens. The *Aspergillus* species was identified from fungal culture plates which had been initially set up on Sabouraud′s 2% dextrose agar (SDA) (Remel, Lenexa, KS, USA) and Inhibitory Mold Agar (Remel, same). *Aspergillus fumigatus* could be identified after 48–72 h of incubation at 35 °C, with identification made by macroscopic observation of colony morphology and microscopic observation using Lactophenol Cotton Blue dye (Remel, Lenexa, KS, USA). If identification by morphology and stains was not possible from these initial evaluations, specimens were sub-cultured to Potato Dextrose Agar (PDA, Remel, Lenexa, KS, USA) and tested by Matrix-Assisted Laser Desorption Ionization-Time of Flight Mass Spectrometry (MALDI-TOF MS) (Vitek MS, BioMérieux Inc., Durham, NC, USA) wherein the colony from the PDA culture media went through a lysis step before testing. The cells were lysed using an ethanol-acetonitrile-formic acid process to obtain good protein extraction. The cleaned fungal protein was applied to the slide and matrix added following standard procedure. Once dried, the standard protocol was followed for use of the Vitek MALDI-TOF MS to obtain a clean spectrum for evaluation by the MALDI-TOF MS software. Anti-fungal susceptibility testing on the *Aspergillus fumigatus* isolate of patient 2 was performed by the reference laboratory using the broth microdilution method.

### 2.2. Statistical Methods

We conducted descriptive data analyses for all parameters that were collected. These values were reported as frequencies, medians with interquartile range and proportions. All statistical analyses were completed using SPSS software (International Business Machines Corp, New York, USA), version 27.

The study was approved by the institutional review board (IRB #2021002).

## 3. Results

In total, 970 patients were admitted to the ICU for management of COVID-19 infection during the study period of January 2020–March 2021. Among these, respiratory cultures growing *Aspergillus* species were noted in 48 patients. Using the 2020 ECMM/ISHAM consensus criteria (Table 1) for diagnosis of CAPA, 2 patients had proven CAPA, 9 had probable CAPA, and 37 possible CAPA (Supplementary Table S1). The incidence of CAPA in our study was 5%.

Among those with CAPA, 66% of the patients were male with a median age of 67 (IQR 17), ranging from 49–86 years (Table 2). A total of 77% of the patients were white but predominantly of Hispanic ethnicity (88%), followed by Asians (18%) and non-white Hispanics (4%). More than 65% of them had underlying hypertension and diabetes mellitus, and less frequently, other underlying conditions such as renal disease, liver disease, cardiac disease, stroke, and cancer.

The overall severity of illness and mortality was quite high with 100% requiring positive pressure mechanical ventilation, 87% requiring vasopressors for hemodynamic support, and 35% needing new renal replacement therapy. The mean duration of hospital length of stay for the patient group was 30 days and the length of ICU stay was 23 days. Death occurred in 83% of the patients (40/48).

The mean duration from a positive COVID-19 test to *Aspergillus* species being recovered from the respiratory cultures was 16 days, ranging from 4 to 50 days. Preceding the recovery of *Aspergillus* from the cultures, a fever >100.5 was noted in 50% of the patients, and 77% of them had worsening respiratory failure despite adequate support and management. Chest imaging showing cavitation or nodules was seen only in 20% of the patients. Inflammatory markers such as CRP, ferritin, and other parameters such as D dimer and LDH were elevated in most of the patients.

Patients were treated for COVID-19 with remdesivir in 69% of the cases and with steroids in 93%. Dexamethasone was the most used steroid treatment (90%). IL-6 inhibitors such as tocilizumab were not used in any of the patients. Broad-spectrum antibiotics were used in over 90% of the patients for an average duration of 7 days.

The most common type of respiratory culture from which the *Aspergillus* species were isolated was an endotracheal aspirate (Table 3). *Aspergillus fumigatus* was the most common species (52%) isolated, followed by a mixed growth with more than one type of *Aspergillus* species (18%) followed by *Aspergillus niger* (12%). Serum galactomannan was tested in 14 of the 48 patients, and 6 out of those were positive. Broncho-alveolar lavage (BAL) fluid galactomannan was tested in three patients, none of which were positive. Serum 1,3 beta-D-glucan was tested in 25 of the 48 patients, and 60% of them were positive. In over 60% of these patients, a bacterial co-infection in the lungs was not detected.

Antifungals were given for treatment in 44% of the patients for a mean duration of 13 days. Voriconazole was the most used agent (66%) followed by a combination of more than one agent (28%), echinocandin (9%), and posaconazole (4%).

There were two cases of proven CAPA in our study (Table 4). One of the proven cases had anti-fungal susceptibility testing performed on the second isolate recovered during therapy. The minimum inhibitory concentration to voriconazole and isavuconazole was 2 µg/mL (Supplementary Table S2), concerning for resistance.

The second proven case with severe COVID-19 infection requiring mechanical ventilation was clinically worsening and grew *Aspergillus fumigatus* from blood and endotracheal cultures, 12 days after the initial SARS-CoV-2 infection. He expired before the availability of these results and never received any anti-fungal therapy.

**Figure 1:**
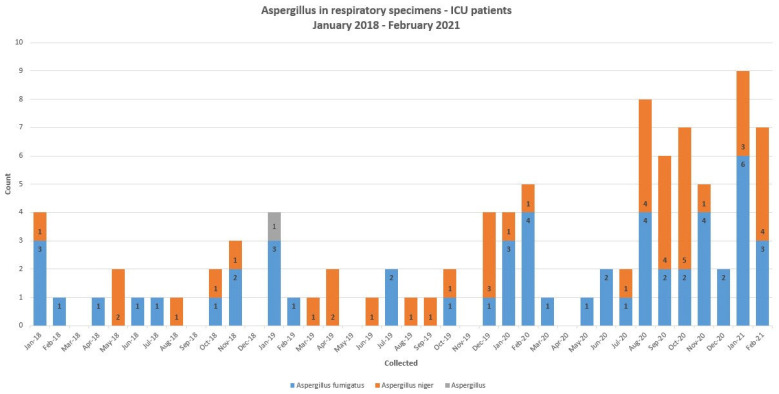
The numbers of *Aspergillus* species recovered from respiratory samples of intensive care unit patients at our center between January 2018 and March 2021.

**Figure 2:**
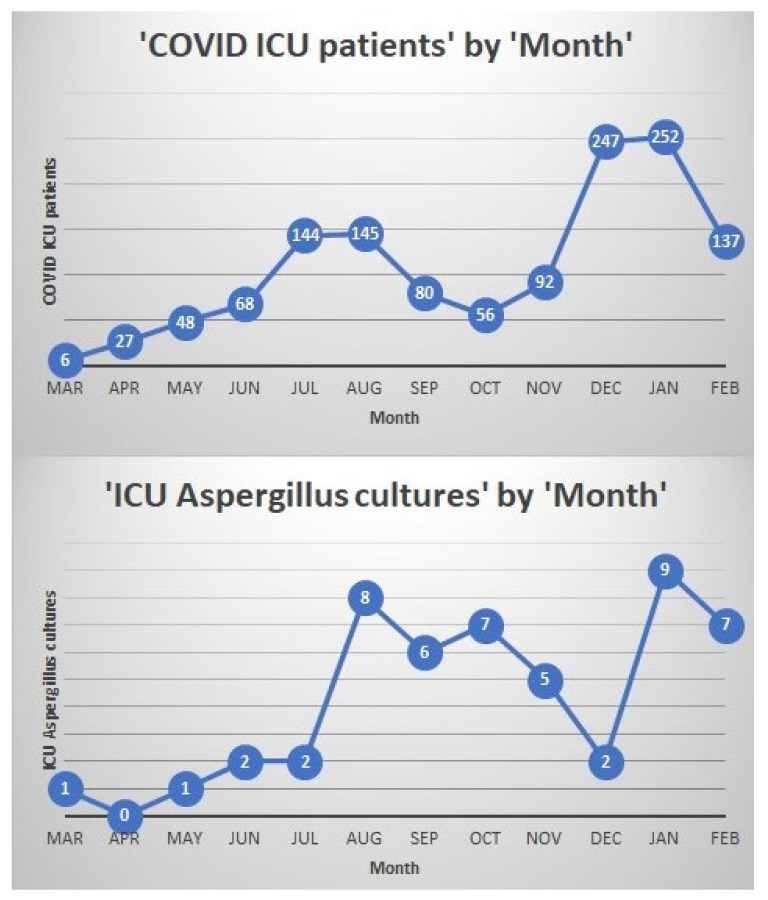
The numbers of COVID-19 patients in the intensive care units and the numbers of *Aspergillus* species recovered from respiratory samples of intensive care unit patients at our center between March 2020 and February 2021.

In the preceding years, in 2018 and 2019, there were 16 and 19 patients with positive *Aspergillus* cultures from respiratory tract specimens from the intensive care units in our center (Figure 1). There was a total of 57 patients in the intensive care units who had positive *Aspergillus* respiratory cultures between January 2020 and March 2021. During this time, the culture positivity was higher during the months of August and September 2020 as well January and February 2021, when there was peak surge of COVID-19 cases in our center (Figure 2).

## 4. Discussion

We describe here a large cohort of 48 patients with CAPA in mechanically ventilated intensive care unit patients at large tertiary care center in Fresno, California, United States of America. The incidence of CAPA in our study was around 5%. Previous studies have shown incidences varying from 3 to 34% [6,7,8,9,10,11,12,13,14,15,16,17,18,19,20,21,22,23,24,25]. Practices in obtaining specimens such as tracheal or endotracheal aspirates, bronchoscopy specimens, fungal biomarkers as well local geographic variations in the incidence of *Aspergillus* likely contributed to these variations. Compared to previous years, the number of *Aspergillus* cultures from respiratory samples did increase significantly during the study period in our center and coincided with the peak surges of COVID-19 infections. However, this number may likely be reflective of the increase in overall ICU bed capacity, mechanically ventilated patients, and cultures obtained during the pandemic.

Definitions using the 2020 ECMM/ISHAM consensus criteria place emphasis on obtaining bronchoscopy specimens for cultures as well as obtaining galactomannan or polymerase chain reaction (PCR) tests for diagnosis. However, bronchoscopies and galactomannan and PCR testing were carried out sparingly in our center. This contributed to most of the patients being placed under the possible rather than probable category.

Sixty-five percent of patients who had CAPA in our study were male, above the age of 65 and Hispanic with underlying hypertension and diabetes. This represents the demographics that were most affected by the COVID-19 pandemic in the region. Although the clinical and imaging features were quite non-specific, half of the patients with CAPA had new or recrudescent fever as well as worsening respiratory failure despite adequate antibiotic management.

Higher minimum inhibitory concentration (MIC) to voriconazole at 2 µg/mL was noted in the *Aspergillus fumigatus* isolate of the patient with biopsy proven CAPA. Gene sequencing was not performed to identify an underlying mutation. This patient did not have any identified EORTC/MSG classic host factors for IPA and developed CAPA around 30 days after initial ICU admission for COVID-19. She also had evidence of *Aspergillus* tracheobronchitis. Cases of voriconazole-resistant CAPA have been reported [28,29,30]. Whether CAPA or the delay in effective anti-fungal therapy due to resistance contributed to her mortality is unclear but a possibility. This adds to the challenges in management of CAPA such as the requirements for therapeutic drug monitoring, neurotoxicity, and hepatotoxicity as well as drug–drug interactions.

Interestingly, the second patient with proven CAPA had *Aspergillus fumigatus* isolated in their blood cultures. This patient’s illness was quite rapidly fatal, as would be expected with invasive disseminated aspergillosis infection. He also did not have any traditional EORTC/MSG risk factors such as neutropenia, immunosuppression, or prolonged corticosteroids. He had severe COVID-19 infection requiring mechanical ventilation and developed IPA 12 days after diagnosis of COVID-19. He never received any anti-fungal therapy as the diagnosis was only made post-mortem. Detection of *Aspergillus* species in blood cultures is quite rare. Previous reports of such an occurrence have been in severely immunocompromised patients such as those with leukemia, transplantation, chronic granulomatous disease (CGD), and acquired immunodeficiency syndrome (AIDS), or in patients with device-related infections and endocarditis [31,32,33,34,35,36,37,38]. *Aspergillus* fungemia in the setting of IPA in the intensive care unit in otherwise non-immunocompromised patients is very unusual. One of the pathological features of SARS-CoV-2 infection seems to be defects in immune cell functions caused by marked hyperinflammation [39]. This resultant reduction in T-lymphocyte numbers as well as their functions is one suggested mechanism for the secondary opportunistic infections seen post-severe COVID-19 infection.

We have here described a large cohort of CAPA cases seen in our center in the past year and more of the pandemic with a rigorous review of patient’s charts. However, our study is limited because it is a retrospective study from a single center. The use of fungal biomarkers, *Aspergillus* PCR, and pursuing bronchoscopies were limited in our study population.

Despite being well reported, CAPA remains an entity with many uncertainties. In addition to standardizing definitions, practices for timely diagnosis such as obtaining bronchoscopy samples and the use of fungal biomarkers must be standardized.

## Figures and Tables

**Table 1:** 2020 ECMM/ISHAM£ consensus criteria for COVID-19-associated pulmonary aspergillosis (CAPA).

	Host Factors	Clinical Factors	Mycological Evidence
Proven	COVID-19 * needing intensive care and a temporal relationship	Pulmonary infiltrate, or cavitating infiltrate (not attributed to another cause)	One of the following: histopathological or direct microscopic detection of fungal hyphae, showing invasive growth with associated tissue damage aspergillus recovered by culture or microscopy, or histology or PCR ** obtained by a sterile aspiration or biopsy
Probable	COVID-19 needing intensive care and a temporal relationship	Pulmonary infiltrate, or cavitating infiltrate (not attributed to another cause)	One of the following: microscopic detection of fungal elements indicating mold in BALƗ positive BAL culture serum galactomannan index >0.5 or serum LFA¥ index >0.5 BAL galactomannan index ≥1.0 or BAL LFA index ≥1.0 two or more positive aspergillus PCR tests in plasma, serum, or whole blood single positive aspergillus PCR in BAL
Possible	COVID-19 needing intensive care and a temporal relationship	Pulmonary infiltrate, or cavitating infiltrate (not attributed to another cause)	One of the following: microscopic detection of fungal elements indicating mold in non-bronchoscopic specimen positive non-bronchoscopic culture single non-bronchoscopic specimen galactomannan index >4.5 or >1.2 twice or more or >1.2 plus another non-bronchoscopic positive mycology test such as PCR

£ European Confederation of Medical Mycology and the International Society for Human and Animal Mycology; * coronavirus disease 2019; ** polymerase chain reaction; Ɨ bronchoalveolar lavage; ¥ lateral flow assay.

**Table 2:** Patient demographics and characteristics of COVID-19 * infection.

Characteristics	*n* (%)
Total	48 (100%)
**Demographics**	
Age in years median with IQR	67 (17%)
Sex (Male)	32 (66%)
Ethnicity (Hispanic)	33 (68%)
**Comorbidities**	
Hypertension	38 (79%)
Diabetes	31 (64%)
Obesity	22 (45%)
Coronary artery disease	17 (35%)
Lung disease	12 (25%)
Renal disease	12 (25%)
Stroke/dementia	11 (22%)
Liver disease	6 (12%)
Cancer	4 (8%)
Severity of COVID-19 *	
Mechanical ventilation	48 (100%)
Pressor support	42 (87%)
Extracorporeal membrane oxygenation	1 (2%)
Renal replacement therapy	17 (35%)
Treatment of COVID-19 *	
Remdesivir	29 (60%)
Hydroxychloroquine	2 (3%)
Steroids	45 (93%)
Convalescent plasma	13 (27%)
**All-cause mortality (death during admission)**	40(83%)

* Coronavirus disease 2019.

**Table 3:** Characteristics of COVID-19-associated pulmonary aspergillosis (CAPA).

Characteristics	*n* (%)
**Type of respiratory culture**	
Endotracheal aspirate	39 (81%)
Bronchoalveolar lavage fluid	9 (19%)
**Type of Aspergillus species recovered**	
*Aspergillus fumigatus*	25 (52%)
*Aspergillus niger*	6 (12%)
*Aspergillus flavus*	2 (4%)
More than 1 species	9 (18%)
Other Aspergillus species	6 (12%)
**Clinical features of aspergillus infection**	
New or recrudescent fever despite appropriate antibiotics for 3 days	25 (52%)
Worsening respiratory failure despite appropriate treatment	37 (77%)
**Imaging features**	
Cavitation	5 (10%)
Nodules	5 (10%)
Other findings (consolidation, ground glass opacities)	38 (79%)
**Biomarkers**	
Serum 1,3 Beta-D-Glucan	13/21 (61%)
Serum Aspergillus galactomannan	6/14 (42%)
Bronchoalveolar lavage fluid Aspergillus galactomannan	0/3 (0%)
**Anti-fungal therapy given**	21 (44%)
**Type of anti-fungal used**	
Voriconazole	14/21 (66%)
More than 1 antifungal	6/21 (28%)
Echinocandin	2/21 (9%)
Posaconazole	1/21 (4%)
**Response to antifungal treatment**	
Defervescence within 72 h	10/21 (47%)
Improvement in oxygen support in 96 h	4/21 (19%)

**Table 4:** Characteristics of patients with proven COVID-19-associated pulmonary aspergillosis (CAPA).

	Patient 2	Patient 44
Age	49	82
Ethnicity	Hispanic	Hispanic
Medical conditions	Obesity, hypertension, diabetes mellitus	Hypertension, coronary artery disease, prostate cancer in remission, diabetes mellitus
EORTC/MSG * risk factors	None	None
Severity of COVID-19 **	Severe ARDS, mechanical ventilation	Severe ARDS, mechanical ventilation
Steroid therapy	Dexamethasone 10 days	Dexamethasone 10 days
Duration between COVID-19 and CAPA ***	30 days	12 days
Clinical features	Worsening fever, hypoxia	Worsening fever, septic shock and multi organ failure
Source of CAPA diagnosis	Biopsy of bronchoalveolar tissue, Bronchoalveolar cultures	Blood cultures, endotracheal aspirate cultures
Bacterial co-infection at the time CAPA	None	None
Imaging finding	Bilateral consolidations with cavitation	Bilateral ground glass opacities
BAL Aspergillus galactomannan	Not tested	Not tested
Serum Aspergillus galactomannan	Not tested	Not tested
1,3, Beta D glucan	Not tested	Not tested
Aspergillus species	*A. fumigatus* and *A. niger*	*A. fumigatus*
Anti-fungal therapy	Voriconazole, posaconazole, caspofungin	None
Anti-fungal resistance	Possible	Not tested
Outcome	Died	Died

* European Organization for Research and Treatment of Cancer/Mycoses Study Group; ** coronavirus disease 2019; *** COVID-19-associated pulmonary aspergillosis.

## Data Availability

Data is available from the corresponding author upon request.

## Supplementary Materials

The following are available online at https://www.mdpi.com/article/10.3390/jof7110948/s1, Table S1: Clinical, radiological, mycological findings in the forty-eight patients and their ECMM/ISHAM CAPA definition categories, Table S2: Antimicrobial sensitivities of Aspergillus fumigatus isolate from patient 2.
