# Supplementary material for: COVID-19-Associated Pulmonary Aspergillosis: A Single-Center Experience in Central Valley, California, January 2020–March 2021
Source: J Fungi (Basel). 2021 Nov 10;7(11):948. doi: 10.3390/jof7110948 (PMC8618928; doi:10.3390/jof7110948)
Supplement: Supplementary file 1 [file jof-07-00948-s001.zip › jof-1415856-table S1.pdf]

Supplemental Table S1: Clinical, radiological, mycological findings in the forty-eight patients and their ECMM/ISHAM CAPA definition categories

Patient	Interval from COVID* to first Aspergillus detection in days	Fever >100.5 without another attributable cause	Worsening respiratory failure without another attributable cause	Imaging findings	*Aspergillus* species recovered	Source of *Aspergillus* culture	Number of occasions isolated	Serum GM** index	BALł GM index	Serum BDG¥	ECMM/ISHAM¶ category
1	11	Yes	Yes	Diffuse nodular opacities	*A. Fumigatus* *A. niger*	Endotracheal aspirate	2	NT£	NT	Positive 308 pg/ml	Possible
2	4	No	Yes	Diffuse nodular opacities	*A. Fumigatus* *A. niger*	BAL, Endotracheal aspirate Lung tissue	4	Positive 0.75	NT	Positive 226 pg/ml	Proven
3	14	No	Yes	Diffuse ground glass opacities	*A. Fumigatus*	Endotracheal aspirate	2	Negative	NT	Negative	Possible
4	21	Yes	Yes	Patchy ground glass opacities with area of focal consolidation	*A. Fumigatus*	Endotracheal aspirate	1	NT	NT	NT	Possible
5	12	Yes	Yes	Diffuse ground glass opacities	*A. flavus*	Endotracheal aspirate	1	Positive 2.71	NT	Positive >500 pg/ml	Probable
6	37	Yes	Yes	Diffuse ground glass opacities	*A. niger*	Endotracheal aspirate	2	Negative	NT	NT	Possible
7	12	No	No	Diffuse nodular opacities	*A. niger*	Endotracheal aspirate	1	NT	NT	NT	Possible
8	21	Yes	Yes	Patchy ground glass opacities with area of focal consolidation	*Aspergillus spp.*	Endotracheal aspirate	1	NT	NT	Positive >500 pg/ml	Possible
9	19	No	Yes	Patchy ground glass opacities	*A. Fumigatus*	Endotracheal aspirate	1	Negative	NT	Positive >500 pg/ml	Possible
10	14	No	Yes	Diffuse nodular opacities	*A. Fumigatus*	Endotracheal aspirate	1	Positive 2	NT	Positive >500 pg/ml	Probable
11	7	Yes	Yes	Diffuse ground glass opacities	*A. Fumigatus*	Endotracheal aspirate	2	NT	NT	NT	Possible

12	11	Yes	Yes	Diffuse ground glass opacities	*A. niger*	Endotracheal aspirate	2	NT	NT	NT	Possible
13	8	Yes	Yes	Patchy ground glass opacities	*A. Fumigatus*	Endotracheal aspirate	1	Negative	NT	NT	Possible
14	15	Yes	Yes	Diffuse ground glass opacities	A. niger	Endotracheal aspirate	3	Positive 1.56	NT	Positive >500 pg/ml	Probable
15	13	No	Yes	Diffuse ground glass opacities	*A. Fumigatus*	Endotracheal aspirate	1	NT	NT	NT	Possible
16	32	Yes	Yes	Cavitary lung lesion	*A. niger*	BAL	1	Negative	NT	Positive 299 pg/ml	Probable
17	11	No	No	Patchy ground glass opacities	*Aspergillus* spp.	Endotracheal aspirate	2	NT	NT	NT	Possible
18	12	No	No	Diffuse ground glass opacities	*A. niger*	Endotracheal aspirate	1	NT	NT	Negative	Possible
19	22	No	Yes	Diffuse ground glass opacities	*A. flavus*	Endotracheal aspirate	1	NT	NT	NT	Possible
20	9	Yes	Yes	Patchy ground glass infiltrates	*A. fumigatus*	Endotracheal aspirate	2	NT	NT	NT	Possible
21	21	Yes	Yes	Diffuse nodular opacities	*A. fumigatus*	Endotracheal aspirate	1	NT	NT	NT	Possible
22	6	Yes	Yes	Diffuse ground glass opacities	*A. fumigatus*	BAL	1	NT	Negative	NT	Probable
23	35	Yes	No	Diffuse ground glass opacities	*A. niger*	Endotracheal aspirate	1	NT	NT	NT	Possible
24	20	Yes	No	Diffuse ground glass opacities	*A. niger*<br>*A. fumigatus*	Endotracheal aspirate	2	NT	NT	Negative	Possible
25	9	Yes	Yes	Diffuse ground glass opacities, Lung nodules	*A. fumigatus*	Endotracheal aspirate	2	Negative	NT	NT	Possible
26	17	Yes	Yes	Diffuse ground glass opacities	*Aspergillus* spp.	Endotracheal aspirate	2	NT	NT	NT	Possible
27	14	No	Yes	Diffuse ground glass opacities	*A. fumigatus*	Endotracheal aspirate	1	NT	NT	Positive 83 pg/ml	Possible

28	14	Yes	Yes	Patchy ground glass opacities	*A. fumigatus*	Endotracheal aspirate	1	NT	NT	NT	Possible
29	43	Yes	Yes	Patchy ground glass opacities	*A. fumigatus*	Endotracheal aspirate	1	NT	NT	NT	Possible
30	21	No	Yes	Patchy ground glass opacities	*A. niger*	Endotracheal aspirate	1	NT	NT	NT	Possible
31	17	No	Yes	Patchy ground glass opacities	*A. fumigatus*	BAL	1	Positive 3.9	NT	NT	Probable
32	7	Yes	No	Diffuse ground glass opacities	*A. fumigatus*	Endotracheal aspirate	1	NT	NT	NT	Possible
33	6	Yes	Yes	Patchy ground glass opacities	*A. fumigatus* *A. niger*	Endotracheal aspirate	2	Negative	NT	Positive 167 pg/ml	Possible
34	24	No	Yes	Focal consolidation, patchy ground glass opacities	*A. fumigatus*	Endotracheal aspirate	1	NT	NT	Positive 203 pg/ml	Possible
35	13	Yes	Yes	Focal consolidation, diffuse ground glass opacities	*A. fumigatus* *A. niger*	Endotracheal aspirate, BAL	2	NT	Negative	NT	Probable
36	4	No	Yes	Diffuse ground glass opacities	*A. fumigatus*	Endotracheal aspirate	2	NT	NT	NT	Possible
37	25	Yes	Yes	Diffuse ground glass opacities	*A. fumigatus* *A. niger*	Endotracheal aspirate	1	Negative	NT	Negative	Possible
38	16	No	Yes	Diffuse ground glass opacities, cavitary lesion	*A. fumigatus*	Endotracheal aspirate	1	NT	NT	NT	Possible
39	50	No	No	Diffuse ground glass opacities	*A. fumigatus*	Endotracheal aspirate	1	NT	NT	Negative	Possible
40	7	Yes	No	Cavitary lung lesion	*A. fumigatus* *A. niger*	Endotracheal aspirate	2	Positive 1.14	NT	Positive 188 pg/ml	Probable
41	9	No	No	Focal consolidation	*A. fumigatus*	Endotracheal aspirate	1	NT	NT	NT	Possible
42	4	No	Yes	Patchy ground glass opacities	*A. fumigatus* *A. niger*	Endotracheal aspirate		Negative	NT	NT	Possible

43	29	No	Yes	Cavitary lung lesion	*Aspergillus species*	Endotracheal aspirate, BAL	2	NT	NT	NT	Probable
44	12	Yes	Yes	Diffuse ground glass opacities with patchy consolidation	*A. fumigatus*	Endotracheal aspirate, blood	2	NT	NT	NT	Proven
45	9	No	No	Focal consolidation	*A. fumigatus*	Endotracheal aspirate	1	NT	NT	NT	Possible
46	12	Yes	Yes	Patchy ground glass opacities	*A niger*	Endotracheal aspirate	1	NT	NT	NT	Possible
47	13	No	Yes	Diffuse ground glass opacities	*A. niger*	Endotracheal aspirate	1	NT	NT	NT	Possible
48	12	Yes	Yes	Diffuse ground glass opacities	*A. niger*	Endotracheal aspirate	1	NT	NT	NT	Possible

*Coronavirus disease 2019 **Galactomannan ł Broncho-alveolar lavage ¥ 1,3, beta-d-glucan ¶ European Confederation of Medical Mycology and the International Society for Human and Animal Mycology £not tested

Reference range for 1,3, beta-d-glucan < 60 pg/ml, serum aspergillus galactomannan index > 0.5, BAL aspergillus galactomannan index > 1.0

Foot note for patient number 2 Tissue biopsy: Numerous fungal organisms morphologically consistent with Aspergillus along with acute inflammation
