# Supplementary material for: COVID-19-Associated Pulmonary Aspergillosis: A Single-Center Experience in Central Valley, California, January 2020–March 2021
Source: J Fungi (Basel). 2021 Nov 10;7(11):948. doi: 10.3390/jof7110948 (PMC8618928; doi:10.3390/jof7110948)
Supplement: Supplementary file 1 [file jof-07-00948-s001.zip › jof-1415856-table S2.pdf]

Table S2 Antimicrobial sensitivities of Aspergillus fumigatus isolate from patient 2

Amphotericin B	2	MIC (µg/ml)
Anidulafungin	≤ 0.06	MEC (µg/ml)
Caspofungin	≤0.06	MEC (µg/ml)
Isavuconazole	2	MIC (µg/ml)
Itraconazole	0.25	MIC (µg/ml)
Micafugnin	≤0.06	MEC (µg/ml)
Posaconazole	≤0.06	MIC (µg/ml)
Voriconazole	2	MIC (µg/ml)

MIC: Minimum inhibitory concentration MEC: Minimum effective concentration
